# Genes Involved in Susceptibility to Obesity and Emotional Eating Behavior in a Romanian Population

**DOI:** 10.3390/nu16162652

**Published:** 2024-08-11

**Authors:** Maria Vranceanu, Lorena Filip, Simona-Codruța Hegheș, David de Lorenzo, Anamaria Cozma-Petruț, Timea Claudia Ghitea, Carmina Mariana Stroia, Roxana Banc, Oana Maria Mîrza, Doina Miere, Vasile Cozma, Daniela-Saveta Popa

**Affiliations:** 1 Department of Toxicology, Faculty of Pharmacy, “Iuliu Hatieganu” University of Medicine and Pharmacy, 6 Pasteur Street, 400349 Cluj-Napoca, Romaniadpopa@umfcluj.ro (D.-S.P.); 2 Department of Bromatology, Hygiene, Nutrition, Faculty of Pharmacy, “Iuliu Hatieganu” University of Medicine and Pharmacy, 6 Pasteur Street, 400349 Cluj-Napoca, Romania; anamaria.cozma@umfcluj.ro (A.C.-P.); roxana.banc@umfcluj.ro (R.B.); oana.stanciu@umfcluj.ro (O.M.M.); dmiere@umfcluj.ro (D.M.); 3 Academy of Romanian Scientists (AOSR), 3 Ilfov St, 050044 Bucharest, Romania; 4 Department of Drug Analysis, Facullty of Pharmacy, “Iuliu Hatieganu” University of Medicine and Pharmacy, 6 Pasteur Street, 400349 Cluj-Napoca, Romania; cmaier@umfcluj.ro; 5 UCL Great Ormond Street Institute of Child Health, 30 Guilford St, London WC1N 1EH, UK; david.delorenzo@ucl.ac.uk; 6 Doctoral Scool of Biomedical Sciences, Faculty of Medicine and Pharmacy, University of Oradea, 1 Universităţii Street, 410087 Oradea, Romania; timea.ghitea@csud.uoradea.ro (T.C.G.);; 7 Department of Parasitology and Parasitic Diseases, Faculty of Veterinary Medicine, University of Agricultural Sciences and Veterinary Medicine of Cluj-Napoca, 3–5, Mănăştur Street, 400372 Cluj-Napoca, Romania; vasile.cozma@usamvcluj.ro; 8 Academy of Agricultural and Forestry Sciences Gheorghe Ionescu-Siseşti (A.S.A.S.), 61, Mărăști Boulevard, 011464 Bucharest, Romania

**Keywords:** genes, *CLOCK*, *GHRL*, *FTO*, *MC4R*, *LEP*, *LEPR*, obesity, emotional eating, diet, precision nutrition

## Abstract

Obesity, a significant public health concern with high prevalence in both adults and children, is a complex disorder arising from the interaction of multiple genes and environmental factors. Advances in genome-wide association studies (GWAS) and sequencing technologies have identified numerous polygenic causes of obesity, particularly genes involved in hunger, satiety signals, adipocyte differentiation, and energy expenditure. This study investigates the relationship between six obesity-related genes (*CLOCK, FTO, GHRL, LEP, LEPR, MC4R*) and their impact on BMI, WC, HC, WHR, and emotional eating behavior in 220 Romanian adults. Emotional eating was assessed using the validated Emotional Eating Questionnaire (EEQ). Our analysis revealed significant variability in obesity-related phenotypes and emotional eating behaviors across different genotypes. Specifically, *CLOCK/*CC, *FTO/*AA, and *LEP/*AA genotypes were strongly associated with higher obesity metrics and emotional eating scores, while *GHRL/*TT and *MC4R/*CC were linked to increased BMI and WHR. The interplay between genetic predisposition and emotional eating behavior significantly influenced BMI and WHR, indicating a complex relationship between genetic and behavioral factors. This study, the first of its kind in Romania, provides a foundation for targeted interventions to prevent and reduce obesity and suggests potential strategies for gene expression modulation to mitigate the effects of emotional eating. Adopting a ‘One Health’ approach by creating an evidence base derived from both human and animal studies is crucial for understanding how to control obesity.

## 1. Introduction

Obesity is one of the greatest problems facing humanity, despite being a preventable condition. Its prevalence is steadily rising, and no nation has been able to stop this trend thus far [1]. Experts and public healthcare systems are concerned about the economic costs associated with treating the comorbidities linked to this pathology. The estimated annual costs of medical care and economic losses are $2 trillion [2] or 2.8% of the global gross domestic product. The World Health Organization predicted that more than 2 billion people were overweight and 650 million people were obese globally prior to the COVID-19 pandemic [3]. Additionally, there has been a discernible rise in childhood obesity. Research shows that during the lockdown times, obesity incidence is on the rise, especially in teenagers [4]. This is probably because of a decrease in physical activity, poor eating habits, and an increase in screen time. Romania’s condition is similar to the worldwide pattern. Obesity rates in Romania and other former communist bloc nations continued to rise alarmingly after the collapse of the Berlin Wall and the end of the communist system in 1989. The PREDATORR study, carried out in Romania from 2012 to 2014, found that 31.9% of people were obese and 34.7% of people were overweight [5]. Experts concur that obesity rates have been rising in Romania over time, despite the fact that no national statistics on the condition were kept during the communist era. According to projections, Romania’s obesity prevalence is expected to reach 50% by 2025, ranking it among the top five European countries with the highest obesity rates [6].

Body Mass Index (BMI) is a commonly used phenotype to describe obesity, which is defined as having an excess of body fat and is a complex combination of genetic, dietary, environmental, behavioral, and psychological factors [7]. In polygenic obesity, which is common in the general population, approximately 600 genes and chromosomal regions have been linked to calorie intake, body composition, and BMI by means of genome-wide association studies (GWAS) [8,9,10]. Only around 25% of these genes linked to obesity susceptibility have, however, been independently confirmed to yet. Together with measurements of adiposity (body fat percentage, waist circumference, and BMI), these genes now comprise a comprehensive list of candidates for obesity susceptibility [11,12,13,14].

Energy intake is a key point in body weight control and several studies have been demonstrated the interaction between genes polymorphism and diet [15,16].

Due to the lack of published data in the field of nutrigenetics and nutrigenomics in our country, increasing obesity among adults and children and the increasing popularity of genetic testing, we thought that is essential to start investigating the genetic implications of emotional eating and body weight in a Romanian population. This research focuses on the examination of emotional eating related polymorphism and links with obesity in order to comprehend how genetic modifications that we considered are related to BMI and obesity susceptibility. Obesity and eating behavior are highly complex traits and for our study the following genes and SNPs were chosen, the reason being the fact that this SNPs were analyzed in numerous studies for gene-diet interactions not simply for the association with obesity. Gene-diet SNPs gives more information about what to do to avoid obesity, and for reducing weight—specific nutritional advices as reduces fat intake, refined carbohydrates, etc. Therefore based on a literature review, the six of the most promising polymorphic sites localized within obesity-related genes were analyzed: Circadian locomotor output cycles kaput (*CLOCK)*, Fat mass and obesity-associated (*FTO*), Ghrelin and obestatin prepropeptide (*GHRL*), Leptin (*LEP*), Leptin receptor (*LEPR*), and Melanocortin-4 receptor (*MC4R*), all characterized in Table 1 [17,18,19,20,21,22,23,24,25] to get a preliminary assessment regarding the influences of genetic factors on obesity and emotional eating.

*CLOCK* gene is a positive regulatory component of the circadian system. Several physiological and pathological situations have been linked to changes in this complicated system. Variation in the *CLOCK* gene have been linked to an increased risk of obesity, type 2 diabetes, mood and sleep disorders, and several forms of cancer [26,27]. The polymorphism rs1801260 (T > C) in the *CLOCK* gene has been linked to several behavioral and physiological changes, including reduced sleep duration, increased energy intake, frequent snacking, skipping breakfast, higher BMI, and greater abdominal obesity [28,29].

The *FTO* gene was identified as an important locus harboring common variants with an unequivocal impact on obesity predisposition and fat mass at the population level is being highly expressed in the hypothalamus, which controls food intake and energy expenditure. Further animal research on mice and pigs provide more evidence in favor of the association studies linking *FTO* mutations to obesity [30]. The *FTO* variant in our study, rs9939609 (T > A) it has been repeatedly demonstrated to linked to greater BMI in a variety of population. There is evidence to support the significance of *FTO* in food cravings, given its widespread expression in the hypothalamus [31].

The *GHRL* gene encodes the hormone ghrelin secreted by the stomach and able to stimulates growth hormone release, hunger, and food consumption [32,33]. The rs696217 (G > T) polymorphism in the *GHRL* gene has been associated in numerous studies with specific dietary patterns, higher fat intake, binge eating behavior, obesity, and larger waist circumferences [22,26].

Leptin was discovered in 1994 by Zhang et al. as the result of the obese gene (*ob*) in genetically obese mice [34]. The most frequently studied polymorphism in the *LEP* gene is rs7799039 (G > A), which is correlated with increased caloric intake, higher BMI, larger waist circumferences, and a heightened risk of obesity [35,36]. Louis Tartaglia and associates discovered the leptin receptor gene in the mouse chromosome 4 db locus in 1995 [37].

Leptin receptors belong to the class I cytokine receptor superfamily. An uncommon obesity condition with mendelian inheritance occurs in humans when the leptin receptor gene (*LEPR*) is mutated. On the other hand, interactions between genes and environment—primarily nutrition—cause obesity in humans. Body weight regulation may be influenced by variations at the *LEPR* locus. The rs1137101 (A > G) polymorphism of the *LEPR* gene was associated with obesity, high leptin level due to disruption of *LEPR* signaling and greater waist circumferences [38].

In 1998 human genetic research revealed that monogenic obesity can result from mutations in the *MC4R* gene [39]. The *MC4R* is a G-protein coupled receptor with a central role in appetite regulation, essential to the leptin–melanocortin pathway [40]. The hypothalamus is the primary site of expression for the protein *MC4R*, which is implicated in the regulation of food behavior, satiety, and energy homeostasis.

*MC4R*’s gene polymorphism, rs17782313 (T > C), was associated with increased energy intake, especially fat intake, uncontrolled eating in women, size of portion and weight gain [41,42]. As it follows from what was said previously several studies have linked these SNPs to obesity-related traits such as BMI, total body weight, hip circumference, waist circumferences, body fat percentage and waist hip ratio, among others and also seem to be associated with eating behavior.

The aim of this study was to analyze the role of these genes polymorphisms individually in the increasing obesity and emotional eating risk and investigate the interplay between genetic variability, emotional eating behavior, and obesity-related physical indicators as a purpose to establish in the future strategy to be included in precision nutrition programs for wellbeing and weight management.

## 2. Materials and Methods

### 2.1. Study Design and Samples

A cross-sectional study of the association between the gene variants mentioned above eating behaviour and obesity in adults was performed.

For this study, were selected subjects who voluntarily attended two weight loss clinics in Romania, for issues related to diet, weight and lifestyle. All the participants in the study were Romanian nationality their relatives being in the territory for many generations. Among the subjects who volunteered, 18 were excluded based on the following criteria: type 2 diabetes, liver diseases, chronic kidney diseases, depression, anxiolytic treatments, diagnosed cancer, inappropriate age, under 18 or over 75.

After exclusion, 220 subjects, men and women, normal weight, overweight and obese were enrolled in the study. Participant data were codified to ensure anonymity. All procedures were carried out in accordance with the norms of good clinical practice. The participants received detailed information about the research, the purpose of the study and the applied procedures.

### 2.2. Anthropometric Measurements

The height was measured using Harpenden digital stadiometer, the participants being barefoot and positioned upright with the head in a normal relaxed position. Weight was measured using the TANITA MC-780 device (Tanita Corporation Japan, Tokyo, Japan) with an accuracy of 0.1 kg according to the procedure specified in the instruction manual (without shoes, without metal objects, lightly dressed).

Body fat distribution of participants was assessed by measurement of waist circumference at the level of the umbilicus and hip circumference with the greatest circumference over the greater trochanters. These values were used to calculate the waist to hip ratio. To the end of the procedure the following parameters were noted: BMI (kg/m^2^), height (cm), weight (kg), waist circumferences (cm), hip circumferences (cm), waist to hip ratio (WHR).

### 2.3. Emotional Eating Measurement

Emotional eating was assessed by a validated Emotional Eating Questionnaire (EEQ) [43], translated into Romanian language, which was given to the subjects, to be fill, at the beginning of the study. This is a self-reported questionnaire and consist of 10 items which have the role of evaluating whether the emotions affect eating behavior.

Examples of the items are: “Do you eat when you are stressed, angry or bored?” ‘‘Do you feel less control over your diet when you are tired after work at night?’’, “How often do you feel that food controls you, rather than you controlling food?” All questions have four possible responses: never, sometimes; generally, and always. Each answer was marked with a score from 0 to 3, the lowest number representing a healthy behavior. Based on the score obtained from EEQ subjects were classified into four groups: (1) scores between 0–5, nonemotional eater; (2) scores between 6–10, low emotional eater; (3) scores between 11–20, emotional eater; and (4) scores between 21–30, very emotional eater.

### 2.4. Genetic Analysis

Buccal cells were collected from the lateral wall of the oral vestibule using the commercial Buccal swab with Rapidri Pouch Isohelix kit, following the manufacturer’s recommendations. The samples were processed in a Clia and Cap accredited laboratory based in UK. DNA was extracted from the buccal cells collected on swabs-a standard method for BEK-3/BEK-50, followed by genotype analysis using the ABI7900 real-time thermocycler system, Waltham, MA, USA, optimized for use with Applied Biosystems chemistry, including nucleic acid quantification and detection. To elaborate, 10–50 ng of genomic DNA was amplified in a total volume of 10 μL. TaqMan Genotyping Mastermix (Applied Biosystems, Waltham, MA, USA), 0.5 μL of 20× TaqMan respective genotyping assay, and 10 μL purified water were used. The PCR conditions were as follows: 10 min at 95 °C (initial denaturation), followed by 40 cycles alternating at 95 °C for 15 s (denaturation) and 60 °C for 60 s (annealing/extension) on a rapid real-time thermocycler Applied Biosystems 7900HT (Applied Biosystems, Waltham, MA, USA). The genotypes were analyzed using the Sequence Detection Systems (SDS) software, version 2.2.1 (Applied Biosystems, Waltham, MA, USA). The following SNPs were analyzed: *CLOCK*—rs1801260 (T > C), *FTO*—rs9939609 (T > A), *GHRL*—rs696217 (G > T), *LEP*—rs7799039 (G > A), *LEPR*—rs1137101 (A > G), *MC4R*—rs17782313 (T > C) [44].

### 2.5. Statistical Analysis

Statistical analysis was conducted using SPSS software (version 20), which involved a comprehensive examination of various parameters such as frequency intervals, mean values, and standard deviations. Tests of statistical significance were employed to determine the reliability of the results. Specifically, the Student’s t-test was used to compare the means of two independent groups. In this context, “t” represents the t-statistic, which assesses whether there is a significant difference between the means of the two groups. For comparisons involving three or more independent groups, the ANOVA (Analysis of Variance) test was applied, with “F” denoting the F-statistic. This test determines whether there are any statistically significant differences between the means of the groups. If the ANOVA indicated significance, the Bonferroni post hoc test was subsequently used to perform pairwise comparisons between the groups to identify specific differences. The Bravais-Pearson correlation coefficient was calculated to measure the strength and direction of the linear relationship between two continuous variables. This coefficient ranges from −1 to 1, with values closer to 1 or −1 indicating stronger correlations. In the analysis, a *p*-value of less than 0.05 was considered statistically significant, indicating that the results are unlikely to have occurred by chance. A *p*-value of less than 0.01 was considered highly significant, suggesting a stronger evidence against the null hypothesis. These thresholds helped determine the validity and reliability of the observed associations and differences among the studied groups.

Deviations from Hardy-Weinberg equilibrium of genotype frequencies at individ-ual loci were assessed using standard χ^2^ tests.

## 3. Results

The characteristics of the studied population are shown in the Table 2.

The dataset comprised measurements from 220 individuals, including 88 men and 132 women were aged 18–69 years old. The average height was 170.35 cm (SD = 8.04), and the average weight was 76.38 kg (SD = 15.70), leading to an average BMI of 26.18 (SD = 4.53). Waist and hip circumferences averaged at 83.63 cm (SD = 14.41) and 101.16 cm (SD = 7.41), respectively, resulting in an average WHR of 0.82 (SD = 0.10). Emotional eating behavior, as assessed by the questionnaire, revealed an average score of 6.59 (SD = 3.67).

We calculated the Hardy-Weinberg Equilibrium (HWE) for six genes (*CLOCK, GHRL, FTO, LEP, LEPR, MC4R*) related to obesity, using genotype frequencies from our dataset [45]. The results are shown in the Table 3.

This table provides a comprehensive overview of the Hardy-Weinberg equilibrium analysis results, including both the probability of observing the data if the population is in equilibrium (*p*-value) and the chi-squared statistic, which quantifies the difference between observed and expected genotype frequencies.

The analysis of HWE revealed significant deviations in *GHRL, LEP,* and *LEPR* genes, whereas *CLOCK, MC4R*, and *FTO* genes appeared to be in equilibrium.

The genotype markers distribution inside the Romanian population is shown in the Table 4.

The analysis of six genes revealed a genotype-phenotype correlation, subjects carrying *CLOCK*/CC genotype displayed the highest average weight 95.27 kg, BMI 32.63, WC 101.10 cm, HC 108.88 cm and WHR 0.93, *FTO*/AA genotype average weight of 95.44 kg, BMI of 31.85, WC of 102.21 cm, HC 108.76 cm, WHR at 0.94, *LEP*/AA average weight of 95.13 kg, BMI 32.73, WC 101.41 cm, HC 108.95 cm and WHR 0.92, categorizing all of them into the obese range.

The less affected by obesity were *CLOCK/*TT genotype, average weight, 69.33 kg, BMI 24.07, WC 76.37 cm, HC 97.91 cm, WHR of 0.78 and *FTO*/TT, average weight 66.58 kg, BMI 23.32, WC 73.99 cm, HC 96.89 cm, WHR 0.76.

For the other three genes there is a correlation with overweight and obesity especially for the genotypes *GHRL*/TT, with an average weight of 87.15 kg, BMI of 30.00, waist circumference 94.38 cm, HC 103.58 cm and waist/hip ratio 0.90. *LEPR*/GG average weight of 80.33 kg, BMI of 27.64, and WC of 88.49 cm, HC 103.48 cm, WHR 0.84 and *MC4R*/CC average weight of 82.65 kg, BMI 28.93, WC 82.10 cm, HC 99.87 cm, WHP at 0.81 indicate a trend towards higher body fat and central obesity.

Regarding *CLOCK*/CT, *FTO*/AT, *GHRL*/GT, *LEP*/GA, *LEPR*/AG, *MC4R*/CT we found intermediates values for weight, BMI, waist circumference, and WHR, reflecting moderate levels of body fat and obesity.

The mean value of BMI according to each genotype is shown in the Table 5.

**Figure 1:**
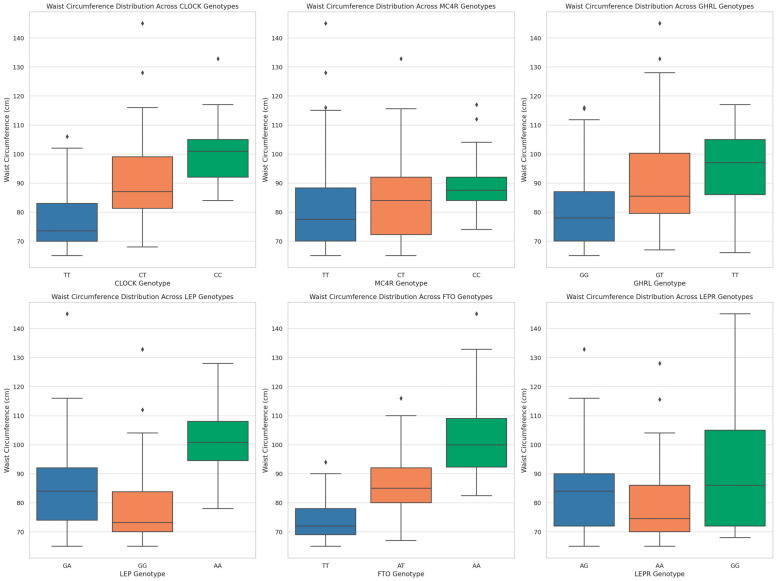
Waist circumference differences across different genotypes for each gene.

Waist circumferences differences across different genotypes for each gene are illustrated in the Figure 1.

Regarding the Emotional eating score (EES) values we found that emotional eaters are represented by *GHRL*/TT genotype carriers, EES 11.38, SD 3.86 and *CLOCK*/CC, EES 11.12, SD 2.82, the highest among the genotypes. The less affected by emotional eating issues were the individuals carrying *FTO*/TT, genotype, with EES 4.73, SD 2.45, *CLOCK*/TT, EES 4.90, SD 2.49, *LEP*/GG, EES 5.02, SD 2.49, *GHRL*/GG, EES 5.54. EES differences across different genotype are shown on the Table 6.

**Figure 2:**
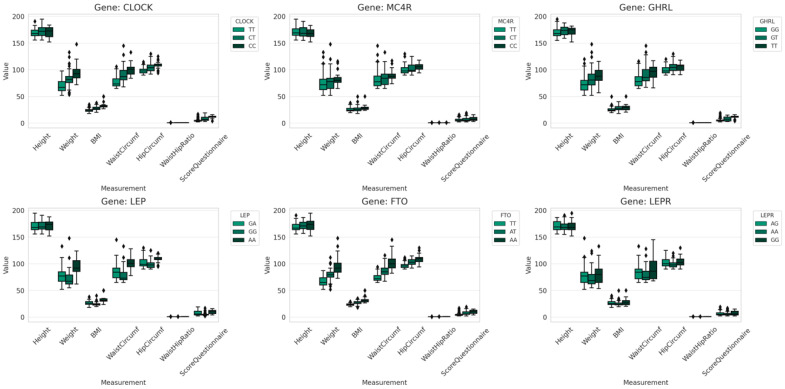
The distribution of physical measurements and emotional eating scores across the different genotypes for each of the six genes (*CLOCK, FTO, GHRL, LEP, LEPR, MC4R*).

In Figure 2 is represented the distribution of physical measurements and emotional eating scores across genotypes. This comprehensive view highlights the variability present in the dataset and can be used to infer potential genotype-phenotype associations, such as differences in weight, BMI, waist circumference, hip circumference, waist/hip ratio, and emotional eating scores among the different genotypes of each gene.

These visualizations allow for an immediate comparison of central tendency, variability, and outliers across genotypes within each gene for all measured parameters.

It is also investigated the interplay between genetic variability, EES, and obesity-related physical indicators BMI and WHR to understand whether and how genetic predispositions and behavioral patterns contribute to obesity, both independently and in combination. The results showed a significant independently effect of *CLOCK* gene and EES on BMI and WHR and also a significant effect of the interaction *CLOCK* genotypes-EES (F = 5.468, *p* < 0.001); (F = 3.711, *p* = 0.0061).

**Figure 3:**
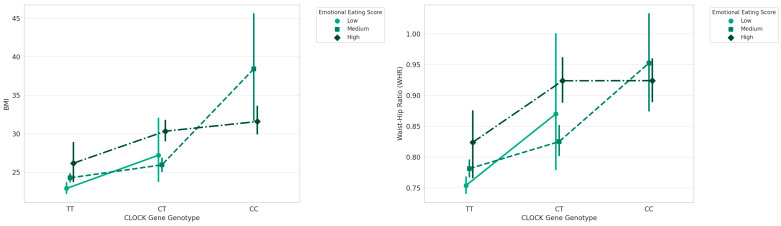
Interaction between *CLOCK* genotype and EES on BMI and WHR.

The effect of the *CLOCK* genotype and EES on WHR results highly significant (F = 17.869, *p* < 0.00001); (F = 63.210, *p* < 0.00001) and the effect of interaction *CLOCK* genotype and EES is significant (F = 3.711, *p* = 0.0061) Figure 3.

**Figure 4:**
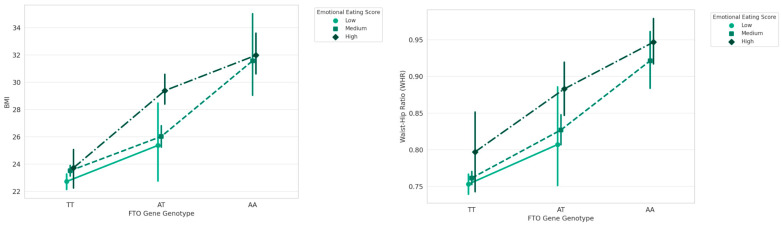
Interaction between *FTO* Genotype and Emotional Eating Scores on BMI and WHR.

*FTO* genotypes and EES shown a highly and respectively an extremely significance on BMI (F = 82.843, *p* < 0.00001), (F = 200.306, *p* < 0.00001) and a significant effect for the interactions *FTO* genotypes-EES (F = 13.839, *p* < 0.00001). The effect of *FTO* genotype and EES on WHR are highly significant (F = 50.906, *p* <0.00001), (F = 141.629, *p* < 0.00001) and the interaction effect is significant (F = 11.434, *p* < 0.00001), Figure 4.

*GHRL* genotypes (show a borderline significance (F= 2.350, *p* = 0.0978), on BMI indicating a marginal influence of this gene on BMI variability while the effect of EES is highly significant (F = 48.358, *p* < 0.00001). The *GHRL* genotype-EES on BMI is not significant (F = 1.031, *p* = 0.3924). The effect of the *GHRL* gene on WHR is not significant (F = 0.868, *p* = 0.4212), indicating no substantial influence of the *GHRL* gene on WHR variability but the effect of EES on WHR is highly significant (F = 38.395, *p* < 0.00001), indicating a strong influence of emotional eating behavior on WHR. The interaction *GHRL* genotype -EES on WHR is not significant (F = 1.177, *p* = 0.3219). These results indicate that while emotional eating scores have a significant impact on WHR, there is no significant interaction between these scores and the *GHRL* genotype on WHR. Additionally, the *GHRL* gene itself does not have a significant effect on WHR.

The *MC4R* genotypes and EES effects on BMI, are significant (F = 6.831, *p* = 0.0013) and (F = 55.050, *p* < 0.00001) but the interaction *MC4R* genotype-EES on BMI is not significant (F = 1.115, *p* = 0.3505). These results indicate that while the *MC4R* genotype and emotional eating scores independently have significant effects on BMI, there is no significant interaction between these two factors. *MC4R* genotypes shows a trend towards significance on WHR (F = 2.407, *p* = 0.0926), while the effect of EES is highly significant (F = 41.634, *p* < 0.00001). The interaction between *MC4R* genotype and emotional eating score categories on WHR is not significant (F = 0.549, *p* = 0.7001). The *MC4R* gene itself shows a marginal effect on WHR, which does not significantly vary across different levels of emotional eating behavior.

**Figure 5:**
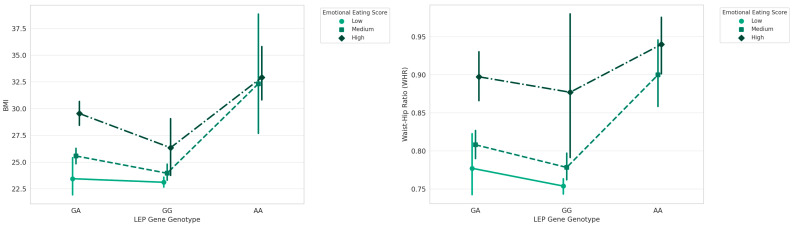
Interaction between *LEP* Genotype and Emotional Eating Scores on BMI and WHR.

Regarding the *LEP* gene and EES we found a statistically significant effects on BMI and WHR for *LEP* genotype (F = 24.960, *p* < 0.00001), (F = 11.781, *p* < 0.00001), highly significance of EES effects (F = 102.573, *p* < 0.00001), (F = 75.162, *p* < 0.00001) and a significant interaction effects (F = 3.438, *p* = 0.0095) but only on BMI ranks (Figure 5).

From the analysis, *LEPR* genotypes and EES alone have significance on BMI ranks but no statistical significance was found regarding the interaction effect. The lack of significant interaction suggests that the effect of emotional eating on BMI does not markedly differ among the *LEPR* gene genotypes. The effect of *LEPR* genotype on WHR is not significant, but the EES is high significant (F = 45.135, *p* < 0.00001) and also the interaction effects is significant (F = 3.349, *p* = 0.011).

**Figure 6:**
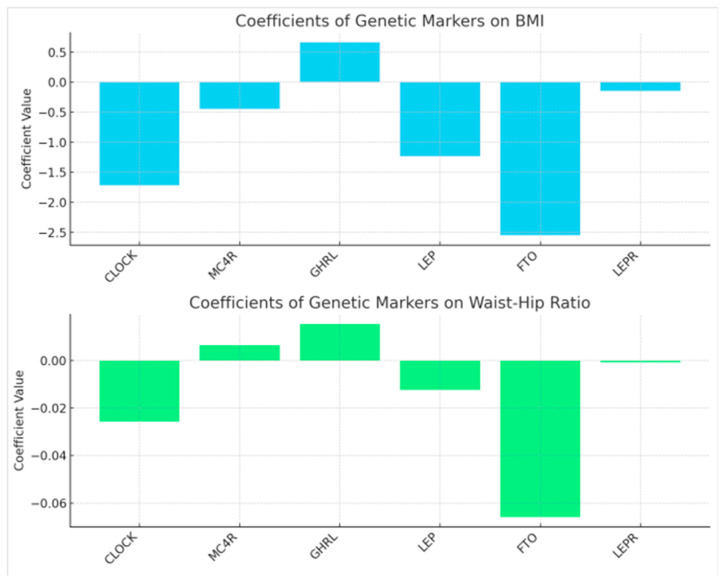
The coefficients of genetic markers on both BMI and Waist-Hip Ratio from the regression analyses. Each bar represents the magnitude and direction (positive or negative) of the relationship between a specific genetic marker and the obesity metric.

To investigate the associations between specific genetic markets, BMI, WC, HC, WHR and obesity metrics we conducted a regression analysis to explore these associations. The results reveal a significant impact of certain genetic markers on both BMI and WHR, underscoring the genetic basis of obesity metrics, Figure 6.

Specifically, regression analysis without controlling for emotional eating revealed significant associations for specific genotypes with obesity metrics. Thereby genotypes *CLOCK*/CC and *FTO*/AA were indirectly associated with higher BMI, *GHRL*/TT showed a direct association and *MC4R*, *LEP,* and *LEPR* genotypes did not show significant associations. The *GHRL*/TT was directly associated with higher WHR, and *FTO*/AA was indirectly associated with higher WHR. Other markers did not exhibit significant relationship.

## 4. Discussion

In this study, we examined the alleles and genotype distributions of the *CLOCK*-rs1801260 (T > C), *FTO*-rs9939609 (T > A), *GHRL*-rs696217 (G > T), *LEP*-rs7799039 (G > A), *LEPR*-rs1137101 (A > G), and *MC4R*-rs17782313 (T > C) polymorphisms in a Romanian adult population. Our aim was to determine if there is a correlation between these genotypes and obesity, and to assess how different genotypes influence individual eating behavior and the extent to which this behavior correlates with obesity.

Our statistical analyses revealed that specific genotypes are associated with increased anthropometric measurements such as BMI, WC, HC, WHR and emotional eating behavior. Notably, individuals with the *CLOCK* (CC), *FTO* (AA), and *LEP* (AA) genotypes exhibited the highest BMI and WHR, while those with *GHRL* (TT), *MC4R* (CC), and *LEPR* (GG) genotypes, although showing lower values, were still correlated with overweight and obesity. Carriers of the *CLOCK* C, *FTO* A, *LEP* A, *GHRL* T, *MC4R* C, and *LEPR* G alleles had a higher likelihood of increased BMI and WHR. This observation represents the first important finding of this study, showing that certain specific alleles can be unfavorable for some individuals predisposing to a higher risk of obesity and related disorders. Given that WC and WHR are useful predictors of abdominal obesity [32], this study highlights the potential role of the *CLOCK, FTO*, and *LEP* gene variants in increasing the risk of this type of obesity, which is often associated with cardiovascular disease. The results are consistent with other studies conducted on European and non-European populations, adult and children [23,46]. The mechanisms by which genes influence the risk of obesity are different. The variation in anthropometric measurements and EES across *CLOCK* genotypes indicates a range of body weights highlighting potential genetic influences on weight regulation and suggest a genetic component to how individuals emotionally respond to food, potentially linked to circadian rhythms and their regulation. The results of interaction *CLOCK* genotypes-EES indicate a significant interaction between these two factors, suggesting that the effect of one factor on BMI and WHS depends on the level of the other factor. This implies that the relationship between EES, BMI and WHR varies across different *CLOCK* gene genotypes, and the impact of emotional eating is modulated by the *CLOCK* gene genotype. The significance of the interaction terms supports hypothesis that the genotype and emotional eating scores together influence BMI and WHR and this influence is not merely additive but involves a complex interplay between these factors. Circadian clocks regulate several key biological processes being involved in the control of eating behavior, energy balance, glucose and lipid homeostasis and consequently the regulation of body weight. Various clock genes are involved in the maintenance of this mechanism, *CLOCK*, being one of nine main proteins expressed for this purpose.

Alterations in *CLOCK* gene, can interfere with this complex mechanism and leading to multifactorial diseases. The *CLOCK* T > C polymorphism has been associated with obesity, sleep duration, response to weight loss. The carrier of C allele seems to be at a higher risk for obesity, showing elevated BMI and WHR, high EES, higher energy intake and less responsive to weight loss intervention [47] and sleep duration [48]. Studies have found that poor sleep disrupts metabolism, increases cravings and hunger by disrupting hunger hormones like ghrelin, increasing the level of cortisol, a stress hormone and decreasing the insulin sensitivity [49].

The *CLOCK* C allele has been also associated with abnormal circadian rhythm, delayed breakfast time, evening preference, lower satiety and low compliance to mediterranean diet [50]. Emotional eating is usually associated to less weight loss and one important factor to explain this observation is disinhibition, a type of eating behavior characterized by a reduction in the ability to control the calories ingested and therefore the subject is prone to high caloric intake. The *CLOCK* CC genotype interacts with disinhibition, an important driver for this gene-environment interaction, associated with a high anthropometric measurement and obesity.

Also, it appears that the circadian system not only influences lipid metabolism, but increasing evidence shows that fatty acids might, in turn, regulate various chronobiological functions. Garaulet et al. (2010) [51] utilising 1100 participants from the GOLDN (Genetics of Lipid Lowering Drugs and Diet Network) study, concluded that there were different effects in *CLOCK* T > C genotypes for saturated fat intake: at an intake ≥11.8% of total energy, C allele carriers showed larger waist circumferences compared to non-carriers, while an intake <11.8% of total energy did not lead to significant differences between carriers and non-carriers in terms of waist circumference. All these aspects related to how different *CLOCK* genotypes can influence or not weight gain, sleep duration, emotional eating, diet adherence, are extremely important for the healthcare practitioner, dietician, doctor, in order to implement a personalized diet program based on genotype. Considering the fact that the carriers of the C allele have a poor diet adherence the use of nutraceuticals can also be considered in order to alter gene expression, restoring the normal epigenetic profile and aiding weight loss. Thereby, supplementation with Epigallocatechin-3-Gallate showed beneficial effects on circadian misalignment, obesity, insulin resistance, and lipid metabolism by normalizing the expression of the clock genes *CLOCK* and *BMAL1*, while resveratrol normalizes the rhythm of *CLOCK* and *PER2* in addition to *BMAL1* in animals fed an obesogenic diet [52,53].

The analysis of the *FTO* gene in this study, reveals a clear gradient effect where the AA genotype is associated with the highest levels of obesity and emotional eating behaviors, whereas the TT genotype is linked to the leanest body compositions and the least severe emotional eating patterns. The AT genotype represents an intermediate phenotype, illustrating the dose-dependent effect of the *FTO* gene’s allelic variations on obesity and related behaviors. These findings underscore the *FTO* gene’s pivotal role in modulating body weight, fat distribution, and emotional responses to food. The marked differences across genotypes reinforce the importance of considering genetic predispositions in obesity research and highlight the potential for genetic screening to inform personalized interventions for obesity and its psychological components.

Thereby, in several studies the *FTO* variants, especially the risk allele A of common T/A polymorphism (rs9939609) is linked to poor satiety, increased appetite, a higher intake of dietary fat or protein, overeating, poor eating habits and food choices [23]. The presence of this allele increases the risk of overweight and obesity by 20–30% and it was demonstrated, the people carrying this allele usually eat daily ~350 Kcal more than non-carriers [54]. Subsequent research using various methodologies frequently verified the link between the *FTO* risk polymorphism and decreased satiety. For example, the A-allele of the SNP rs9939609 was linked to considerably lower satiety in a study using psychometric measurement of eating behaviors through questionnaire in 3337 children in the United Kingdom [55].

To data, the molecular mechanism to explain unequivocally the association between *FTO* and obesity risk is not well established. *FTO* gene regulates ghrelin level, the hormone that stimulates appetite, through the methylation of ghrelin mRNA. In the risk allele carriers *FTO* activity is increased and as consequence these carriers has increased ghrelin expression due to decreased ghrelin mRNA methylation [56]. Recently it was found that *FTO* can influence *IRX3* expression, a protein involved in obesity and the browning of adipose cells resulting that carriers of protective *FTO* T allele has lower *IRX3* expression and lower adipose tissues than AA homozygotes [57]. Also *FTO* A it was correlated with a middle emotional eating in our study.

Identifying and understanding the genotypes that lead to overeating are extremely important in weight management. People carrying risk variant can benefit more from high-protein diets, according to data from the Pounds Lost Trial, a 2-year randomized intervention trial that tested the effect of four diet types, varying in proportions of carbohydrates, protein, and fat on weight loss in overweight and obese individuals. The researchers found that *FTO* risk variant carriers lost more weight when they followed a high-protein diet than a low-protein diet. In contrast, for non-carriers subjects a low-protein diet was better [58].

The conclusion is that for carriers of two *FTO* copies of risk variants, protein intake up to 20% of total daily calories is more effective for weight loss [59], meanwhile the non-carrier, should keep protein intake at 15% of total daily calories for more effective weight loss. For people carrying only one copy of risk variant the protein level does not matter.

The *GHRL* gene, implicated in hunger signaling and regulation of energy balance, shows significant genotype-phenotype associations in our dataset where the TT genotype is associated with higher obesity metrics and emotional eating scores. In contrast, the GG genotype is linked to leaner body compositions and lower emotional eating behaviors, with the GT genotype occupying an intermediate position. These results underscore the potential influence of the *GHRL* gene on obesity and emotional eating, providing insights into the genetic underpinnings of these complex traits. The marked differences across genotypes reinforce the importance of genetic screening in understanding individual susceptibility to obesity and related behavioral patterns, potentially guiding personalized intervention strategies. A major mediator in controlling energy balance and metabolism, the orexigenic hormone Ghrl is mostly released by the stomach in humans being a key mediator in regulating energy balance and metabolism that act on the hypothalamic nuclei by integrating signals from environment that indicate the availability of food and the start and end of the eating episode. Interaction between these elements determines meal portion, meal composition and food intake. Many studies have been associated *GHRL* rs696217 T allele with BMI-related obesity, metabolic syndrome, binge eating [60,61], our results being aligned with the results in the literature.

*LEP* gene encodes a protein secreted by white adipocytes and playing an important role in energy homeostasis regulation. Circulating leptin binds to the leptin receptor in the brain and activates downstream signaling pathways to promote energy expenditure and inhibit feeding. Polymorphisms in the *LEP* gene are linked to obesity and type 2 diabetes development. Many studies have been conducted on the relationship between LEP variants and obesity, one of the most studied variant being LEP rs7799039.

In our study the weight distribution for *LEP* rs7799039 genotypes could be associated with leptin’s role in satiety signaling and energy expenditure, affecting body weight, and variability in waist circumference for *LEP* genotypes could be linked to leptin’s role in energy homeostasis and fat storage, affecting abdominal fat accumulation. The variability in emotional eating scores for *LEP* genotypes might be related to leptin’s role in energy regulation and satiety, affecting emotional responses to food. The most affected by obesity and emotional eating were the carriers of AA genotype an GA. For *LEP* GG genotype we found the lowest average weight and BMI suggesting a leaner body composition and the lowest waist circumference and waist/hip ratio indicative of lesser central obesity.

However, in the literature, there is conflicting evidence amongst different ethnicities regarding the associations between *LEP* G > A polymorphism and obesity. Wang et al. found that in a Taiwanese aboriginal community, subject with GG genotype (wild-type, reference sequence) had higher BMI than those with GA or AA [62]. Similarly, researchers from Turkey, Tunisia, Serbia, Spanish, reported no association between the *LEP* G > A polymorphism and higher BMI [63,64,65,66]. Instead, other studies found a strong association *LEP* AA, GA and obesity [67].

Very interesting, a study conducted in Romania, in 2010, by Constantin A. et al. did not find any association between *LEP* G > A and obesity risk, the researchers concluding that *LEP*/2548GG genotype appear to be important only in regulating leptin levels [68]. A Romanian retrospective study, with a limited number of participants, published recently, analyzed the relationship between *LEP* rs7799039 and found that the carriers of *LEP* GG genotype were characterized by normal BMI while the carriers of GA genotype were overweight or obese [69]. This aspect is very important showing the need for other controlled, qualitative studies to understand how genetics impact on individual’s weight and how to manage the weight loss programs in case of obesity.

The *LEPR* rs1137101 analysis highlights a gradient effect in which the GG genotype is associated with higher obesity metrics and emotional eating scores, whereas the AA genotype is linked to leaner body compositions and lower emotional eating behaviors. The AG genotype occupies an intermediate position, showcasing the nuanced influence of *LEPR* gene variations on body composition and eating behaviors.

These findings underscore the role of the leptin signaling pathway, mediated through the *LEPR* gene, in influencing obesity and related behaviors. The differences across genotypes suggest that individual genetic variations can significantly impact susceptibility to obesity and emotional eating, offering insights for personalized approaches to managing these conditions. The analysis of the *LEPR* gene, along with other obesity-related genes, underscores the complexity of genetic contributions to obesity and highlights the importance of considering genetic profiles in obesity research and intervention strategies. *LEPR* encodes the leptin receptor and is considered a candidate gene for susceptibility to leptin resistance, people with less sensitive leptin receptors would be more likely to be overweight and to develop resistance. Some researchers say the opposite, that highly sensitive leptin receptors may lead to leptin resistance. Leptin suppresses appetite and increases the fulness feeling and the desensitized of leptin receptor lead to leptin resistance and weight gain.

Many *LEPR* variants are currently under investigation for their possible link to leptin resistance and obesity, the most important are rs1137100 and rs1137101. *LEPR* rs1137101, the GG genotype is associated with higher BMI, increased calories intake, higher cholesterol and blood sugar level and insulin resistance.

The overeating, a major driving force behind obesity, is largely coded in genes responsible for regulation of appetite and satiety and *MC4R* is one of these. *MC4R* codes for the protein melanocortin 4 receptor, which is found predominantly in paraventricular nucleus of the hypothalamus, an area responsible for controlling appetite and satiety, the *MC4R* being a critical regulator of energy homeostasis (intake and expenditure). Several polymorphisms of the *MC4R* gene have been reported to be associated with overeating and obesity. The polymorphism rs17782313 located downstream of *MC4R*, has been associated with overweight and obesity in many studies. The minor allele C of this polymorphism is correlated with reduced *MC4R* expression, compared to the major allele T.

Analysis of genetic data from 77,228 European adults concluded that each copy of the C-allele at this polymorphism increases by 8% the risk for being overweight. Carriers of C allele eat more, snack more frequently foods with high fat content and have low satiety. The results of our study regarding the effect of C allele on body weight, are aligned with results from other studies. Thus, the *MC4R* CC genotype was associated with overweight and based on the EES they result low emotional eater. Few studies found that *MC4R* CC genotype is associated with obesity only in women and Horstmann et al. who found an increased EES among the C allele carriers suggested that the effect of *MC4R* CC genotype on eating behavior is mediated by central mechanisms that are sex-specific [70]. We did not have a sex stratification, and this is a serious reason to continue our research on these data especially because more research is needed to establish a definitive link between *MC4R* CC genotype and sex-specific obesity risk.

This finding seems to be interesting and notable to be deepen analyzed in another study to clear understand the mechanism beyond these effect interaction in order to find solutions for obesity prevention and treatment program. If we are looking to the genes related to obesity and analyzed in this study many of them are expressed particularly in the hypothalamus having a key role in regulating food intake and energy. More and more scientific evidence show that genetic factors play an important role in individual differences regarding eating behavior and in term of inherent vulnerability to environment people are not equal. Researchers say that subjects with a genetic susceptibility to emotional eating might benefit from interventions that teach emotions and other strategies to improve emotional status and weight loss. An aspect to be taken into consideration would be the assessment of eating behavior using validated questionnaires. The EES is the validated measure that is most frequently used to examine emotional eating in clinical samples. These questionnaires are all restricted to evaluating negative emotions alone. But happy feelings have been associated with emotional eating time and time again, especially in non-clinical groups. Furthermore, a cross-cultural comparison is necessary to guarantee that a translated questionnaire has the same qualities and operates in the same manner as the original measure, functioning as intended in a different sample

The use of precision nutrition to optimize the dietetic intervention based on individual genetic profile represents a valuable tool for dietitian and patient too. It is true that multi-omics technologies, have changed healthcare practices by acknowledging that every person is different and that individualized dietary recommendations are necessary. People may benefit more from a customized diet based on their biology thanks to recent advancements in precision medicine and nutrition. This field of customized nutrition, which integrates genetic data into clinical practice and is expanding quickly, presents a significant challenge to medical professionals, particularly nutritionists.

It is important to note that as this field of study is very new, many ethical issues and a number of moral dilemma and concerns must be resolve in order to produce sound knowledge that the general public can comprehend. Further research is needed to deeply understand the role of gene polymorphism in obesity and eating behavior.

Likewise, it is of interest to perform bibliographic and experimental studies regarding the complex interaction between genetic predisposition and behavioral factors, in the vision of the concept “One Health, one Medicine, one Welfare” through comparative genetic evaluations, taking into account the swine species, in free range growing conditions, exposed to some microbiological and parasitic contaminants. Because pigs and humans are biologically comparable enough for study, swine have been used in biomedical research much more frequently in the past ten years [71].

The Porcine Translational Research Database is a collection of genes and proteins that can be compared with those primarily studied in humans and rodents. The researchers studied the impact of nutrition on immune and inflammatory responses at pigs in a way that aims to promote both human and animal health. Through the measurement of metabolic alterations in pig tissues and biofluids of pigs fed a high-fat diet, some study has examined the potential of using pigs as models for human obesity investigations.

Juvenile pigs were studied as a model for childhood obesity, which is a crucial area of research due to the challenges of assessing metabolic abnormalities in obese children. The researchers investigated metabolic changes in the intestinal tissue, pancreas, brain, and liver of the pigs [72]. Examining these organ tissues allows for the identification of alterations that could indicate disease or an inadequate response to diet.

The limitations of this study are represented by the lack of diversity in terms of geographic location, generally the participants, all Romanians, were located in the west and south of Romania, the majority from the cities of Cluj-Napoca, Bucharest, Târgu Mureș and Brașov and they all came from the urban environment. Another limitation, the selection, was random in order of presentation to the nutrition clinic but might be selection bias considering their approach towards a weight management and lifestyle changes program. The study has a cross-sectional design determining associations without determining the cause and for this reason other longitudinal studies, randomized controlled trials are necessary to confirm our finding regarding the genotype distribution obesity and eating behavior in the Romanian population. Our cross sectional study it should constitute the start for other quality studies in this field.

## 5. Conclusions

This is the first and a unique study In Romania to analyze the relationship between genetic factors, obesity and eating behavior and the first Romanian study to consider six related obesity genes together. The results, association with obesity, role of some polymorphisms in EES, effects of both genes genotype and EES on BMI and WHR ranks highlight the intricate relationship between genetic factors, obesity, eating behavior, revealing a complex interplay and the need for further research in this area.

Nutrigenomics teaches us that certain food compounds, certain biomolecules can change gene expression and in this way the knowledge of these compounds but also of the individual genetic profile can be an advantage in the fight against obesity. The emphasis will move to prevention in the future. Finding genetic predispositions among family members who are at risk and most likely in the general population will be simpler, even routine. People who are at risk will therefore need to be made aware of the benefits of leading healthy lives, minimizing risk, and pursuing preventive treatments. In order to profit from advances in our understanding of gene-nutrient interactions, society will need to establish or make use of suitable social, ethical, legal, educational, and economic frameworks. When genomic discoveries, such gene-nutrient disease connections, are ready to be assessed as potential instruments to enhance health screening and suggest dietary values, public health and regulatory systems will need to be established. In conclusion, findings from the current study have possible implications for both future research and clinical practice.

## Figures and Tables

**Table 1:** Characteristics of the studied genes and polymorphic sites.

Gene	Chromosome Location	Gene Product	Variant	Function of Protein
*CLOCK*	4q12	A basic helix loop-helix-PAS transcription factor	rs1801260; 3111 T > C	Sleep disorders, metabolic syndrome, obesity, emotional eating
*FTO*	16q12.2	2-oxoglutarate (2-OG) Fe (II) dependent demethylase	rs9939609; T > A	Control of daily food intake, appetite, and satiety-overeating control, nutrients preferences
*GHRL*	3p25–26	Ghrelin-obestatin prepropeptide	rs696217 G > T	Obesity, eating disorders, metabolic syndrome
*LEP*	7q32.1	Leptin	rs7799039; G > A	Regulates appetite, increases in energy expenditure, body composition
*LEPR*	1p31.3	Leptin receptor	rs1137101; A > G Gln223Arg	Mediating leptin signaling
*MC4R*	18q21.32	Melanocortin 4 receptor	rs17782313; T > C	One of the major regulator of food intake and energy expenditure

**Table 2:** Characteristics of the participants.

Gender	Count	Height (cm) (±SD)	Weight (kg) (±SD)	BMI (kg/m^2^) (±SD)	Waist Circumferences (cm) (±SD)	HIP Circumferences (cm) (±SD)	WHR (±SD)	SQ (±SD)
MEN	88	178.42 ± 5.02	86.09 ± 14.84	26.91 ± 3.69	92.10 ± 14.42	104.38 ± 7.20	0.88 ± 0.10	7.38 ± 3.85
WOMEN	132	164.97 ± 4.29	69.92 ± 12.66	25.69 ± 4.97	77.97 ± 11.34	99.01 ± 6.76	0.78 ± 0.07	6.07 ± 3.47

**Table 3:** Overview of the Hardy-Weinberg equilibrium analysis results.

Gene	*p* Value	Chi-Squared Statistic
*CLOCK*	0.120592	4.230683
*FTO*	0.369244	1.992597
*GHRL*	0.028898	7.087998
*LEP*	0.000005	24.439422
*LEPR*	0.008863	9.451751
*MC4R*	0.099333	4.618563

**Table 4:** Genotype distribution inside the population.

Genes	Genotype	Distribution (*N*)
*CLOCK* rs1801260; 3111 T > C	TT	128
	CT	66
	CC	26
*FTO* rs9939609; T > A	TT	110
	AT	64
	AA	46
*GHRL* rs696217 G > T	GG	147
	GT	60
	TT	13
*LEP* rs7799039; G > A	GG	23
	GA	111
	AA	86
*LEPR* rs1137101; A > G	AA	70
	AG	101
	GG	49
*MC4R* rs17782313; T > C	TT	124
	CT	70
	CC	26

**Table 5:** Impact of each genotype on BMI (mean value).

Genes	Genotype	BMI Mean (±SD)	N	F (ANOVA)	*p*
*CLOCK*	TT	24.07 ± 2.67	128	50.041	0.001
	CT	27.88 ± 3.75	66		
	CC	32.63 ± 5.86	26		
*MC4R*	TT	25.53 ± 3.85	124	5.085	0.002
	CT	26.50 ± 5.13	70		
	CC	28.93 ± 5.13	26		
*GHRL*	GG	25.14 ± 3.77	147	14.441	0.001
	GT	27.90 ± 4.60	60		
	TT	30.00 ± 7.33	13		
*LEP*	GG	23.96 ± 2.75	86	37.833	0.001
	GA	26.68 ± 3.68	111		
	AA	32.73 ± 6.17	23		
*FTO*	TT	23.33 ± 1.78	110	125.922	0.001
	AT	27.00 ± 3.17	64		
	AA	31.85 ± 4.95	46		
*LEPR*	GG	27.64 ± 5.39	49	4.401	0.013
	AG	26.17± 4.02	101		
	AA	25.17± 4.36	70		

**Table 6:** Distribution of EES among genotype.

	Gene	Genotypes	EES (±SD)
Emotional Eaters	*GHRL*	TT	11.38 ± 3.86
	*CLOCK*	CC	11.12 ± 2.82
Low Emotional Eaters	*CLOCK*	CT	8.13 ± 3.85
	*MC4R*	CT	6.94 ± 3.67
		TT	6.25 ± 3.66
		CC	7.81 ± 3.81
	*GHRL*	GT	8.13 ± 3.74
		GG	5.54 ± 3.04
	*LEP*	GA	7.16 ± 3.83
		AA	10.04 ± 3.72
	*FTO*	TA	7.25 ± 3.59
		AA	10.13 ± 3.36
	*LEPR*	AG	6.38 ± 3.54
		GG	7.45 ± 3.70
		AA	6.30 ± 3.81
Non-Emotional Eaters	*LEP*	GG	5.02 ± 2.49
	*CLOCK*	TT	4.90 ± 2.49
	*GHRL*	GG	5.54 ± 3.04
	*FTO*	TT	4.73 ± 2.45

## Data Availability

The original contributions presented in the study are included in the article, further inquiries can be directed to the corresponding author.
